# Omalizumab for the treatment of patients with severe allergic asthma with immunoglobulin E levels above >1500 IU/mL

**DOI:** 10.1016/j.waojou.2023.100787

**Published:** 2023-06-10

**Authors:** Francesco Menzella, Jocelyne Just, Inessa Schwab Sauerbeck, Claudia Mailaender, Fabiana Saccheri, Celine Thonnelier, Xavier Jaumont, Laurence Mala

**Affiliations:** a Head, Pulmonology Unit, S. Valentino Hospital, AULSS 2 Marca Trevigiana, Italy; b Allergology Department, Trousseau Hospital, AP-HP Paris, France; c Allergology Department, Université Paris Sorbonne, AP-HP Paris, France; d Novartis Pharma GmbH, Nürnberg, Germany; e Novartis Farma S.p.A., Milan, Italy; f Novartis Pharma S.A.S., Rueil-Malmaison, France; g Novartis Pharma AG, Basel, Switzerland

**Keywords:** Immunoglobulin E, Omalizumab, Severe allergic asthma, Dosing

## Abstract

Immunoglobulin E (IgE) plays a critical role in the allergen-initiated inflammatory pathway and thus serves as a viable therapeutic target in allergic or IgE-mediated diseases such as asthma. Omalizumab, an *anti*-IgE biologic, has been approved in the United States (US, 2003) and in the European Union (EU, 2005) as an add-on therapy in patients with moderate-to-severe persistent asthma and severe allergic asthma (SAA) aged 6 years and older. The dose and frequency of omalizumab are adjusted based on the patient's body weight and baseline IgE levels, as recommended by its dosing tables. Currently, these dosing recommendations are limited to patients with baseline IgE levels of up to 1500 IU/mL in the European Union and 700 IU/mL in the United States. However, many patients with SAA have IgE levels >1500 IU/mL, highlighting an unmet need. This review presents the current evidence on the treatment benefits of omalizumab in patients with IgE levels >1500 IU/mL. The findings from the reviewed studies which included >3000 patients support the efficacy and effectiveness of omalizumab in reducing exacerbations, and improving asthma control, lung function, and quality of life in patients with severe asthma having IgE levels beyond the current dosing range. Omalizumab was well-tolerated in these patients, with no new safety signals. In addition, high IgE levels (>1500 IU/mL) are also reported in several comorbidities of asthma (allergic rhinitis, atopic dermatitis, allergic bronchopulmonary aspergillosis [ABPA], food allergy, and nasal polyposis) and omalizumab has demonstrated efficacy and safety in these indications. These data suggest that omalizumab may be considered for administration in SAA patients, with high IgE levels outside the current dosing tables. A detailed assessment of patients with high IgE levels is needed before deciding on the optimal treatment approach. A management algorithm for SAA patients with IgE >1500 IU/mL is proposed in this review and a suggestion to follow the Delphi consensus is advised.

## Introduction

Severe asthma is one of the most common chronic respiratory disease affecting children and adults worldwide. It is estimated that 3–10% of the global population of 350 million patients with asthma have severe asthma.^1^, ^2^, ^3^

Asthma is a heterogeneous disease, with allergic asthma being 1 of the most common phenotypes in asthmatic patients, especially in children with severe asthma.^4^^,^^5^ Nearly 50% of patients with severe asthma have allergic asthma.^6^ Allergic asthma is characterized by type 2 inflammation, which is triggered by allergens and leads to the synthesis of 1 of the key inflammatory mediators, immunoglobulin E (IgE). Treating severe allergic asthma (SAA) with *anti*-IgE therapy (omalizumab) has a proven efficacy but is limited due to the dosing table of this medication, to a specific range of body weight and baseline blood total IgE levels (maximum level of total IgE of 1500 IU/mL in Europe). This limits the use of omalizumab in SAA patients with total IgE >1500 IU/mL, who are sometimes in great need for such treatment.

The main focus of this review is to discuss the efficacy and safety of omalizumab in patients with asthma with IgE levels >1500 IU/mL. In addition, the article provides an overview on the role of IgE in allergic inflammation, IgE levels in asthma, the rationale for dosing considerations of omalizumab in asthma and a proposed approach for clinical management for these patients. The efficacy of omalizumab in other diseases with high IgE levels is also discussed.

### Role of IgE in allergic inflammation

IgE plays an important role in allergic diseases such as asthma, atopic dermatitis, and food allergy; it is also known to play a role in non-allergic diseases such as nasal polyposis and chronic spontaneous urticaria.^7^ Exposure to an allergen prompts the dendritic cells to present the processed antigens to the *naïve* T-cells, which subsequently differentiates into allergen-specific Th2 cells. This process consequently leads to B cell-mediated release of IgE, specific to the allergen, which binds to the high-affinity IgE receptor (FcεRI) on mast cells, basophils, and even dendritic cells.^8^^,^^9^ Re-exposure to the same allergen initiates cross-linking of bound IgE, and downstream effects such as mast cell degranulation and release of histamine, tryptase and other mediators occur within seconds to minutes of allergen exposure.^10^ A late reaction occurs when the release of pro-inflammatory cytokines and other soluble factors by mast cells stimulates the migration of eosinophils, Th2 cells, basophils, and leukocytes to the inflammatory site. Thus, IgE plays an essential role in both the early and late phases of the allergic cascade and consequently the pathogenesis of allergic diseases.^7^^,^^11^, ^12^, ^13^

### IgE levels in asthma

IgE levels are generally low in plasma (∼0.4 IU/mL)^14^ and serum (<100 IU/mL)^15^ of healthy adults. In allergic diseases, levels of IgE are in most cases significantly higher. A study in Spain reported that total IgE levels were nearly four-fold higher in patients with allergy compared with healthy subjects (204.29 *vs* 46.65 IU/mL).^14^

Patients with asthma reported increased IgE levels compared to individuals without asthma.^16^, ^17^, ^18^, ^19^ Moreover, IgE levels may also differ in patients with varying severity of asthma. One study reported IgE levels of 464 IU/mL in patients with mild asthma and 1045.32 IU/mL in those with severe asthma;^18^ similar findings were reported in the TENOR and other studies.^20^, ^21^, ^22^ Although studies generally report median IgE levels of approximately 100 IU/mL to 300 IU/mL in patients with severe asthma, it has been documented that some patients in these studies had IgE levels of 1000 IU/mL or greater (up to 3000 IU/mL).^18^^,^^23^^,^^24^ Other studies on SAA showed that many patients had higher levels of IgE (up to 68,628 IU/mL).^25^, ^26^, ^27^, ^28^, ^29^, ^30^, ^31^, ^32^, ^33^

The elevation of IgE in asthma has also been observed in children. In a retrospective chart review conducted in 70 children (mean age, 8.8 years), 12% of children with asthma had IgE levels >2000 IU/mL.^34^^,^^35^ A study based on cluster analysis of inner-city children with severe asthma in the United States reported elevated median total serum IgE levels of 733 IU/mL.^36^ A study conducted in children from Paris, France, with persistent asthma reported patients with high IgE ranging between 657 and 952 IU/mL.^37^

### Anti-IgE therapy

**Fig. 1:**
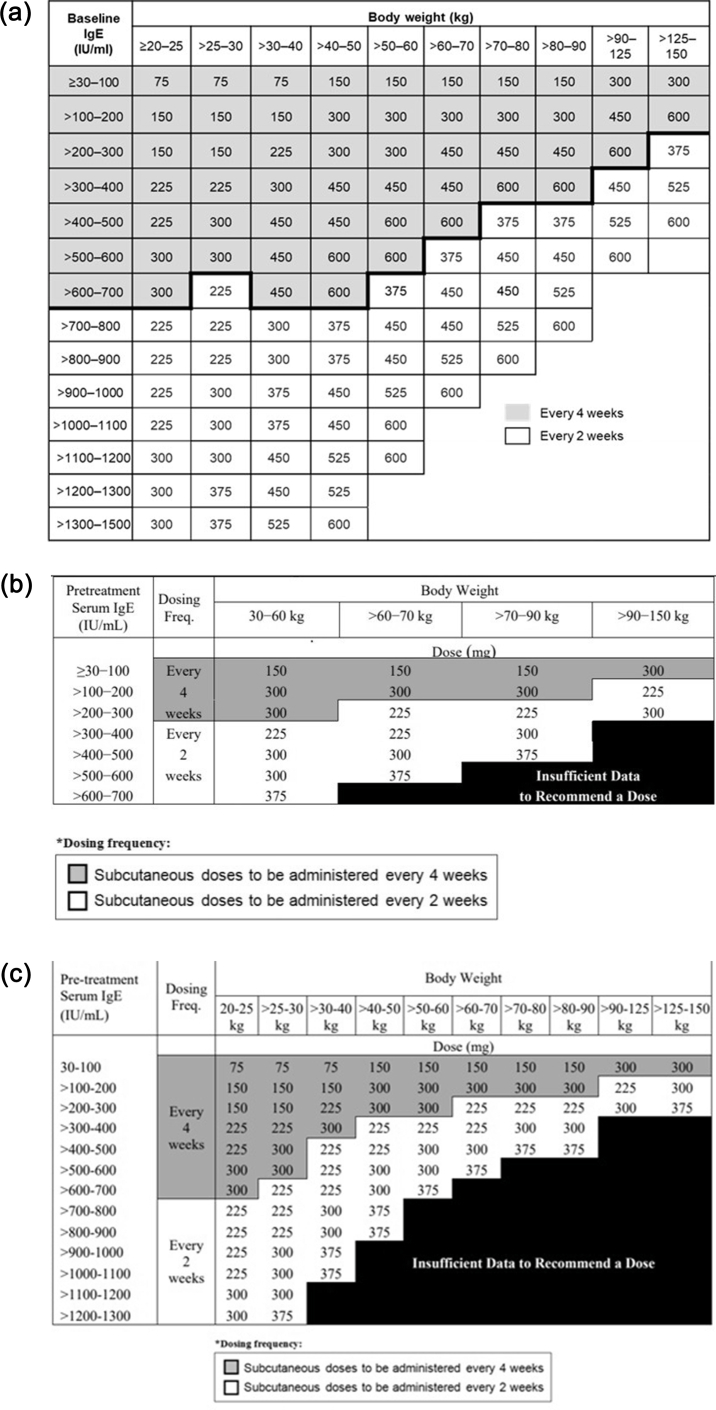
Omalizumab dosing tables (mg/dose). a. EU dosing table in patients aged ≥6 years. b. US dosing table in patients aged ≥12 years. c. US dosing table in patients aged between 6 and <12 years. EU, European Union; IgE, immunoglobulin E; US, United States

Omalizumab is the only approved *anti*-IgE monoclonal antibody in patients aged ≥6 years with uncontrolled moderate-to-severe (United States) and severe (European Union) allergic asthma as an add-on to standard-of-care treatment with high-dose inhaled corticosteroid/long-acting beta-agonist (ICS/LABA).^38^^,^^39^ Omalizumab functions by binding to free IgE at the Cε3 domain, the recognition site of the FcεRI receptor, inhibiting its interaction with the effector cells, thus playing a crucial role in reducing both early- and late-phase allergic reactions.^40^^,^^41^ Omalizumab has a patient exposure surpassing 1.99 million patient-years.^42^ Its therapeutic effect has been established for more than 20 years through numerous clinical trials and real-world studies.^40^ Such data support the efficacy of omalizumab in terms of reductions in asthma exacerbations, asthma-related hospitalizations, and emergency room visits, with improvements in asthma control and lung function.^40^^,^^43^ Omalizumab has also demonstrated a potent oral corticosteroid (OCS)-sparing effect, which can help mitigate the side effects that are associated with the OCS use.^44^ Omalizumab is administered subcutaneously every 2 or 4 weeks, with the dose and frequency adjusted based on the total serum IgE level at baseline and the body weight of the patient, through standardized dosing tables (Fig. 1).

### Rationale for dosing

Initial studies with omalizumab showed that upon administration, it had a very fast effect (within 1 h), reducing free serum IgE by 84%–99% post-single dose.^45^ The dosing strategy was thus tailored to evaluate the dose of omalizumab that would be essential for a sustained reduction in free IgE. The target average free IgE level with omalizumab therapy is 25 ng/mL (10.4 IU/mL). This ensures that ≥95% of patients achieve a level <50 ng/mL (20.8 IU/mL), which is consistently associated with therapeutic benefit.^46^ In order for omalizumab to cause a sufficient reduction in IgE levels (ie. <50 ng/mL), dosing needs to be determined according to patient's baseline IgE level and body weight, at a molar excess of approximately 15-fold over baseline IgE.^47^ The suggested dosing interval for omalizumab is 2 or 4 weeks.^38^^,^^39^

Based on these data, a simplified dosing table for omalizumab was developed based on a range of IgE levels and body weights that were thought to be most prevalent in routine clinical practice.^41^^,^^48^^,^^49^ The initial dosing table for omalizumab approved by the Food and Drug Administration (FDA) and European Medicines Agency (EMA) in 2003 specified a maximum permissible dose of 750 mg every 4 weeks and was applicable to patients with a body weight between 30 and 150 kg and IgE levels of 30–700 IU/mL.^46^ In 2010, based on data from a pharmacokinetic–pharmacodynamic model and evidence from clinical trials, the dosing table for omalizumab was expanded in the European Union and United States.^47^ In European Union, the dosing table was expanded to include patients with body weight from 20 to 150 kg and IgE levels up to 1500 IU/mL with a maximum permissible dose of 600 mg every 2 weeks (Fig. 1).^39^ In the United States, the expanded dosing table included IgE levels up to 1300 IU/mL for patients aged between 6 and <12 years and body weight from 20 to 150 kg, while in adults and adolescents aged ≥12 years, the dosing table is limited to IgE levels up to 700 IU/mL and body weight from 30 to 150 kg, with a maximum permissible dose of 750 mg per 4 weeks.^38^

However, the IgE ranges included in the initial and revised omalizumab dosing tables are limited and many studies have reported patients with SAA having IgE levels outside the dosing table limits, including IgE levels >1500 IU/mL.^25^^,^^26^ For instance, in the INNOVATE study, of the 1006 patients screened, nearly 20% (n = 206) were ineligible for treatment due to baseline IgE levels and body weight being outside the dosing table limits and were excluded (Data on file). In addition, many patients may not have been screened due to the ineligibility as per the IgE levels set in the inclusion criteria. In a pooled analysis, add-on therapy with omalizumab resulted in meaningful treatment benefits in all 4 baseline IgE quartiles (IU/mL: 0–75, 76–147, 148–273, ≥274) across the range of outcome variables. However, efficacy was greater for more outcome variables (including exacerbations) in the upper 3 IgE quartiles, with similar benefits in each of these 3 quartiles.^50^

As guidelines on the appropriate dosing of omalizumab for patients with total IgE>1500 are not available per dosing schedule, many patients may not have received omalizumab making them not eligible to receive omalizumab therapy. We performed a literature review to examine efficacy and safety of omalizumab in studies that have administered the drug outside the current dosing table recommendations.

### Omalizumab use in patients above the recommended dosing limit

**Fig. 2:**
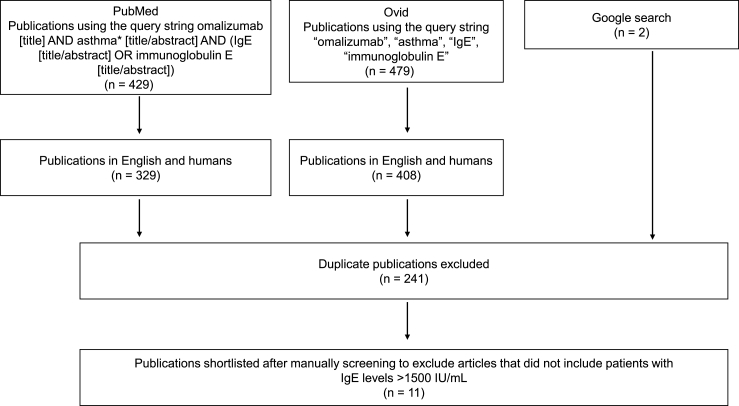
Literature screening. IgE, immunoglobulin E

A literature search was performed on 14 April 2020 (updated on 10 October 2022) in the PubMed and EMBASE databases (accessed through the Ovid database) using the query string “omalizumab AND asthma AND immunoglobulin E/IgE” to compile a list of studies that reported use of omalizumab in patients with high IgE. The literature search was restricted to studies involving humans and publications in English. A Google search was also performed to identify additional literature (Fig. 2). The results were manually screened to select studies of relevance.

The 9 shortlisted studies comprised clinical trials, observational studies and case reports that included patients with SAA with mean IgE levels of up to 68 628 IU/mL.^25^, ^26^, ^27^, ^28^, ^29^, ^30^, ^31^, ^32^, ^33^^,^^51^ The details and main results from each of these studies are presented in Table 1A, Table 1Ba and b and briefly summarized below.

**Table 1A:** Overview of studies on omalizumab in asthmatic patients with high IgE levels.

S.No.	Study	Study design	Patients	Total IgE levels	Assessments
1.	Braunstahl et al., 2013^30^ (eXpeRience registry)	A 2-year, open-label, observational registry	Patients with uncontrolled, persistent allergic asthma who had initiated omalizumab within the 15 weeks prior to start of the study (N = 943)	Baseline IgE: mean ± SD, 323.1 ± 460.9 IU/mL; range, 8–7670 IU/mL	Effectiveness of omalizumab in terms of: physician's GETE, exacerbation rate, symptoms, rescue medication use, OCS use, healthcare resource utilization including hospitalization, ER visits and unscheduled doctor visits, missed work/school days and safety
2.	Maselli et al., 2013^26^	Retrospective, case-control study from January 2006 to December 2010	Patients aged ≥12 years (N = 120) with moderate-to-severe asthma	Patients were divided into two groups: – Group 1 (n = 26): IgE level >700 IU/mL; mean ± SD, 2371 ± 2389 IU/mL – Group 2 (n = 26): IgE level 30–700 IU/mL; mean ± SD, 221 ± 145 IU/mL	Effectiveness of omalizumab was assessed in the two groups in terms of: FEV_1_, ACT score, episodes of systemic corticosteroid use, emergency department visits
3.	Zielen et al., 2013^25^	Multicenter, randomized, double-blind, parallel group, placebo-controlled study with omalizumab treatment period of 12–14 weeks	Patients with allergic asthma aged between 18 and 65 years with body weight ranging from 40 to 150 kg (N = 50)	Patients were grouped into: – Group 1 (n = 18): 30–300 IU/mL IgE; mean ± SD, 131.4 ± 62.3 IU/mL – Group 2 (n = 16): 700–2000 IU/mL IgE; mean ± SD, 1325.3 ± 312.4 IU/mL – Pooled placebo (n = 16): 8 patients with 30–300 IU/mL and 8 with 700–2000 IU/mL IgE; mean ± SD, 598.5 ± 592.9 IU/mL	Efficacy of omalizumab at 8 and 16 weeks in terms of: early-phase allergic response (maximum percentage drop in FEV_1_ during first 30 min post ABP test); FEV_1_ AUC (30 min after APB); late-phase allergic response (maximum percentage drop in FEV_1_ 3–8 h post ABP test); free serum IgE; FeNO
4.	Hew et al., 2016^28^	A 6-month analysis of Australian Xolair Registry, a multicenter, Investigator Initiated Trial observational study registry	Patients with SAA aged ≥12 years (N = 179)	Patients were grouped into: – Within-dose table range group (n = 124): IgE 30–1500 IU/mL – Above-dose range group (n = 55): IgE >1500 IU/mL	Effectiveness of omalizumab in patients treated above the recommended dosing ranges in terms of: ACQ score, AQLQ score, and FEV_1_Effectiveness analysis in a subgroup of patients in the above-range group due to IgE level alone (n = 19) and due to both IgE level and body weight (n = 23)
5.	Chipps et al., 2017^29^ and Casale et al., 2019^31^ (PROSPERO study)	A US-based, 48-week, multicenter, prospective study on the effectiveness of omalizumab of omalizumab	Patients aged ≥12 years with allergic asthma, who had initiated treatment with omalizumab (N = 806)	Median (min, max) total IgE level at baseline: – Total population (N = 806): 193.3 (1.1, 68,628.0) IU/mL – Adolescents (12–17 years; n = 69): 506.7 (2.9, 68,628.0) IU/mL – Adults (≥18 years; n = 737): 175.4 (1.1, 8659.2) IU/mL	Effectiveness of omalizumab in terms of: exacerbation rate, hospitalizations, lung function, asthma control (ACT score) and safety
6.	Humbert et al., 2018^32^ (STELLAIR study)	A 1-year, multicenter, non-interventional, retrospective, observational study using medical records of French patients treated with omalizumab	Patients aged ≥6 years with severe allergic asthma (N = 872)	Median (range) total IgE level at baseline: – Total population (N = 872): 345.0 (2–8700) IU/mL – Minors (6–17 years; n = 149): 850.5 (22–8700) IU/mL – Adults (≥18 years; n = 723): 285.0 (2–6900) IU/mL	Effectiveness of omalizumab was assessed after 4–6 months of treatment in terms of physician's GETE, reduction in annualized exacerbation rate and combination of both definitions, according to blood eosinophils count. Response to treatment was also described in terms of proportion of patients with excellent/good GETE score and with ≥40% reduction in annual exacerbation rate. Reductions in hospitalization and changes in the use of anti-asthmatic medications were also evaluated during the 12 months of treatment
7.	Wang et al., 2018^33^	Case report of patients with omalizumab, initiated between January 2008 and December 2015	Children and adolescents (<18 years) with inadequately controlled, moderate-to-severe allergic asthma (N = 11)	Mean serum IgE: – 6–12 years (n = 6): 2356 IU/mL (range, 1380–4320 IU/mL) – ≥12 years (n = 5):1569 IU/mL (range, 892–2130 IU/mL)	Effectiveness of omalizumab in terms of corticosteroid use, hospitalizations, emergency department visits, ACT score during 12 months of treatment
8.	Diaz et al., 2016^52^ and Singh et al. 2019^27^ (REALITY study)	An 8-year, real-life, retrospective study	Patients with moderate-to-severe allergic asthma aged between 12 and 65 years (N = 198)	Patients were classified into 2 groups based on IgE level: – Group 1 (n = 131): 30–700 IU/mL – Group 2 (n = 67): >700 IU/mL	Response to omalizumab was assessed in terms of three modules: • M1: complete or marked improvement in asthma symptoms (GETE score) • M2 (≥3 of the following): 50% reduction in asthma exacerbations, steroid bursts, ER visits, hospitalizations; ≥12% and ≥200 mL improvement in FEV_1_, ≥3 point improvement in ACT score • M3: M1 and M2
9.	Sesé et al. 2019^51^	Subgroup analysis of children included in the prospective, monocentric, Severe Asthma Molecular Phenotype Paris cohort, conducted from January 2011 to December 2015	Patients with severe asthma and/or steroid-refractory asthma aged 6–18 years who had been treated with omalizumab for ≥4 months	Patients were categorized into three clusters based on response to omalizumab: – Cluster 1 (n = 16): patients with eosinophilic asthma, abnormal lung function and poor response to omalizumab; IgE, mean ± SD, 792 ± 514 IU/mL – Cluster 2 (n = 11): patients with uncontrolled asthma, tobacco smoke exposure, normal lung function and inconstant response to omalizumab; IgE, mean ± SD, 802 ± 1364 IU/mL – Cluster 3 (n = 18): patients with severe asthma with multiple allergic comorbidities with mostly normal lung function and constant response to omalizumab; IgE, mean ± SD, 1871 ± 2127 IU/mL	• Predictive markers of complete response to omalizumab at 4 months • Complete response was defined as: – Total asthma control with an ACT score >20, *and* – Decrease in exacerbations, *and* – Improvement or no change in lung function (FEV_1_ ≥95%, and/or FEV_1_/FVC ≥85%)

ABP, allergic bronchoprovocative test; ACT, Asthma Control Test; ACQ, Asthma Control Questionnaire; AE adverse event; AQLQ, Asthma-related Quality of Life Questionnaire; AUC, area under the curve; ER, emergency room; FEV_1_, forced expiratory volume in 1 s; FVC, forced vital capacity; FeNO, fractional exhaled nitric oxide; GETE, Global Evaluation of Treatment Effectiveness; IgE, immunoglobulin E; IIT, investigator initiated trials; MCID, minimal clinically important difference; N, total number of patients; n, number of patients in each group; OCS, oral corticosteroid; SAE, serious AE; SD, standard deviation.

**Table 1B:** Efficacy and safety of omalizumab in asthmatic patients with high IgE levels.

c	Study	Key findings
1.	Braunstahl et al. 2013^30^ (eXpeRience registry)	• Omalizumab was effective in 69.9% of patients based on physician's GETE score, assessed at 16 ± 1 weeks • More than half (67.3%) of patients experienced no clinically significant exacerbations by the end of the study compared with 6.8% at the pre-treatment period • At Month 24, symptoms and rescue medication use were reduced by >50% and OCS use was reduced by 14.2% compared with baseline • Healthcare resource utilization was lower at Month 24 (mean ± SD, 0.5 ± 1.28) compared with the pre-treatment period (6.2 ± 6.97) • Mean (±SD) number of days of missed work (1.0 ± 4.66 vs 26.4 ± 49.61 days) and missed school (1.9 ± 5.46 vs 20.7 ± 27.49 days) reduced substantially at Month 24 with omalizumab compared with pre-treatment period • Omalizumab was well tolerated with no new safety signals
2.	Maselli et al. 2013^26^	• Data are presented for omalizumab group 1 (n = 26, IgE level >700 IU/mL) and group 2 (n = 26, IgE level 30–700 IU/mL), respectively A non-significant improvement in mean FEV_1_ pre-omalizumab (2.20 L; 2.45 L) and post-omalizumab (2.46 L; 2.55 L) was observed in both groups • Improvement in asthma control (ACT score) was observed in both groups post-treatment (3.36; 3.66) • There was a comparable reduction in the number of episodes requiring corticosteroid use in both groups (1.62; 1.38) • The number of emergency department visits were reduced in both groups post-treatment (0.73; 0.5) • Omalizumab was well tolerated in both groups with no new safety signals
3.	Zielen et al. 2013^25^	• Data are presented for omalizumab group 1 (n = 18, 30–300 IU/mL) and group 2 (n = 16, 700–2000 IU/mL), respectively • A reduction in early-phase response was observed with omalizumab in both IgE groups vs placebo at Week 8 (*P* = 0.018 and *P* < 0.001) and at Week 16 (*P* = 0.087 and *P* < 0.001) • There was greater improvement in FEV_1_ AUC in both omalizumab groups compared with placebo at Week 8 (treatment effect, 13.5, *P* = 0.010; 15.8, *P* = 0.024) and Week 16 (10.8, *P* = 0.049; 13.4, *P* = 0.041) • In patients with late-phase response ≥15% at baseline, greater attenuation of response from baseline was observed with omalizumab in both omalizumab groups at Week 8 (5.3%; −3.5%) and Week 16 (0.23%; 1.50%) vs placebo • Reduction in free IgE to <50 ng/mL was observed in both omalizumab groups post-omalizumab • FeNO levels were reduced with omalizumab in both groups at Week 16 (7%; 16%) • Omalizumab was well tolerated in both omalizumab groups. No SAEs were related to omalizumab
4.	Hew et al. 2016^28^	• Post-treatment with omalizumab, improvement in asthma control (ACQ) vs baseline was observed in above-range (2.10) and within-range (1.93) groups (both *P* < 0.0001) • The majority of patients in the above-range (72.7%) and within-range groups (64.4%) showed ≥0.5 improvement (MCID) in AQLQ • Pre- (*P* < 0.05) and post-bronchodilator FEV_1_% predicted (*P* = 0.05) improved after treatment with omalizumab versus baseline in the above-range participants • Improvement in pre- (*P* = 0.147) and post-bronchodilator FEV_1_ (*P* = 0.214) with omalizumab vs baseline were comparable between within-range and above-range participants • The subgroup analysis comparing participants above range due to IgE alone (38.2%) vs IgE and weight (60%) demonstrated comparable improvements in ACQ-5, AQLQ and FEV_1_
5.	Chipps et al. 2017^29^ and Casale et al. 2019^31^ (PROSPERO study)	• Omalizumab was associated with a marked reduction in exacerbation rate compared with the 12 months before baseline (0.78 ± 1.37 vs 3.00 ± 3.28; *P* < 0.001) in the overall population – The mean exacerbation rate was also lowered in patients with IgE >700 IU/mL compared with the 12 months before baseline (0.71 ± 1.35 vs 3.18 ± 3.73) • The proportion of patients in the overall population with ≥1 hospitalizations was reduced from 22.1% in the 12 months prior to baseline to 4.0% post-treatment • A mean improvement in pre-bronchodilator FEV_1_ of 40 mL, 170 mL and 30 mL was observed in the total, adolescent and adult populations • Improvement in asthma control (ACQ) was observed 12 months post-omalizumab (mean ± SD change from baseline 4.4 ± 4.9) in the overall population – In patients with IgE >700 IU/mL, a mean (±SD) improvement of 5.0 ± 4.9 was observed from baseline • No new safety signals were reported in the overall population
6.	Humbert et al. 2018^32^ (STELLAIR study)	• Omalizumab was associated with treatment effectiveness in 77.2% of minors and 67.2% of adults based on physician's GETE score (excellent/good) • The annual exacerbation rate was reduced by 60.2% in minors and 48.5% in adults after 4–6 months of treatment, and by 70.4% and 58.6% in minors and adults, respectively, during 12 months of treatment • A ≥40% reduction in exacerbations was observed in 78.5% of minors and 71.1% of adults after 4–6 months of treatment • In total, 67.8% of minors and 58.2% of adults were responders, based on both physician's GETE and reduction in exacerbations • Hospitalization rates were reduced by 73.2% in minors and 72.6% in adults during 12 months of treatment • Omalizumab was associated with a near 50% reduction in dose and use of OCS at 12 months
7.	Wang et al. 2018^33^	• Omalizumab was associated with a 55% reduction in the use of corticosteroids, 60% reduction in emergency department visits, and 72% reduction in hospitalizations • A 46% improvement in asthma control (ACT score, 13 vs 19) was observed post-omalizumab • No AEs were related to omalizumab and no new safety signals were reported
8.	Diaz et al. 2016^52^ and Singh et al. 2019^27^ (REALITY study)	• Response rates were assessed at 16 weeks, 1 year, 2 years and 5 years for M1, M2 and M3 – M1: complete or marked improvement in asthma symptoms (GETE score) – M2 (≥3 of the following): 50% reduction in asthma exacerbations, steroid bursts, ER visits, hospitalizations; ≥12% and ≥200 mL improvement in FEV_1_, ≥3 point improvement in ACT score – M3: M1 and M2 • In the overall population (n = 198), response rates for M1, M2 and M3 respectively at 16 weeks were 61.3%, 60.8%, and 41.8%, at 1 year were 84.8%, 72.2%, and 64.6%, at 2 years were 82.4%, 71.2%, and 63.2%, and at 5 years were 95.1%, 87.8%, and 85.4% at 5 years • After 1 year, M1, M2 and M3 response in Group 1 (IgE: 30–700 IU/mL; n = 101) and Group 3 (>1500 IU/mL; n = 36)^a^ was: – M1 was comparable (82.2% vs 86.1%) between Group 1 (and Group 3 – M2 response was comparatively higher in Group 3 (80.6%) vs Group 1 (68.3%) – M3 response was comparable between Group 1 (61.4%) and Group 3 (69.4%); *P* = 0.083
9.	Sesé et al. 2019^51^	• ACT score, lung function and the number of severe exacerbations were used to assess the response to omalizumab • The majority (89%) of patients in Cluster 3 with high IgE (mean ± SD, 1871 ± 2127 IU/mL) were complete responders to omalizumab

ACT, Asthma Control Test; ACQ, Asthma Control Questionnaire; AE adverse event; AQLQ, Asthma-related Quality of Life Questionnaire; AUC, area under the curve; FEV_1_, forced expiratory volume in 1 s; FVC, forced vital capacity; FeNO, fractional exhaled nitric oxide; GETE, Global Evaluation of Treatment Effectiveness; IgE, immunoglobulin E; IIT, investigator initiated trials; MCID, minimal clinically important difference; OCS, oral corticosteroid; SAE, serious AE; SD, standard deviation.

a Group 2 data (IgE: 701–1500 IU/mL; n = 20) was not available in the published abstract.

The two-year observational eXpeRience registry study involved 925 patients with uncontrolled, persistent asthma who were treated with omalizumab. The study included patients with a range of baseline IgE levels outside the dosing table (8–7670 IU/mL; mean: 323.1 IU/mL); no data on number of patients with IgE >1500 IU/mL included in the study was provided. Overall, omalizumab was associated with reduced exacerbations and OCS use, and improved symptoms and asthma control across all IgE levels.^30^

A retrospective, case-control study (26 patients with IgE >700 IU/mL and 26 with IgE 30–700 IU/mL) found that omalizumab was equally safe and effective in reducing corticosteroid use, asthma symptoms and emergency department visits in patients with severe asthma and IgE levels >700 IU/mL (mean, 2371 IU/mL) than those with IgE levels 30–700 IU/mL (mean, 221 IU/mL). Patients with IgE levels >700 IU/mL received a mean (± standard deviation [SD]) omalizumab dose of 886 (±220) mg/month.^26^

In a fourteen-week randomized clinical trial, patients with asthma were given 450–600 mg omalizumab every 2 weeks, with dose adjusted based on body weight and IgE levels at baseline. Patients with IgE levels between 1200 and 2000 IU/mL were administered 600 mg omalizumab every 2 weeks. Allergen-induced bronchoconstriction and airway inflammation measured by fractioned exhaled nitric oxide (FeNO) were markedly reduced with omalizumab in patients (n = 16) with high IgE levels (700–2000 IU/mL); these patients had a sustained reduction in free IgE levels to <50 ng/mL as early as Week 6.^25^

In Australia, the maximum recommended dose of omalizumab in patients with SAA is 750 mg per 4 weeks, as per standard dosing table (IgE: 30–1500 IU/mL and body weight: 30–150 kg; considered as within recommended dosing criteria patients). However, patients with SAA with IgE >1500 IU/mL and body weight >150 kg (above recommended dosing criteria patients) may also be prescribed with omalizumab at a ceiling dose of 750 mg per 4 weeks. In this analysis of the Australian Xolair® Registry, 179 patients with SAA were analyzed, which included 144 patients within recommended dosing criteria and 55 patients above recommended dosing criteria. Omalizumab was effective in improving asthma symptoms, quality of life, and lung function in patients with SAA above the recommended dosing criteria, with effect comparable to that observed in those within recommended dosing criteria, without the need for dose escalation above 750 mg per 4 weeks.^28^

PROSPERO was a multicenter, prospective study in the United States that evaluated the effectiveness and safety of omalizumab in patients with allergic asthma aged ≥12 years over 48 weeks. Nearly 24% of patients had IgE levels above or below those included in the US-based dosing table, with IgE levels up to 68 628.0 IU/mL in adolescents and 8659.2 IU/mL in adults.^29^ Patients treated with omalizumab reported an improvement in asthma control with reduced hospitalizations and exacerbation rates; the improvements were similar in patients with IgE levels within the dosing table range and outside the dosing table range.^29^

STELLAIR was a one-year, retrospective, real-life study in French patients with SAA who were treated with omalizumab. IgE levels in the study were in the range of 2–8700 IU/mL at baseline. Omalizumab was associated with treatment effectiveness in the majority of patients: ∼70% of patients rated their response to treatment as "excellent"/"good" on the Global Evaluation of Treatment Effectiveness (GETE) scale, while ∼70% of patients had a ≥40% reduction in annualized exacerbation rate.^32^

In a study of 11 children and adolescents being treated at The Children's Hospital of Philadelphia, omalizumab was found to be safe and effective with sustained improvement in asthma control and reduction in hospitalizations in patients with severe asthma and high IgE levels (up to 4320 IU/mL); of note, 7 patients had IgE >1500 IU/mL.^33^ All the patients received omalizumab at a dose of 375 mg every 2 weeks.

In the retrospective REALITY study (131 patients with IgE 30–700 IU/mL and 67 with IgE >700 IU/mL), ∼70% of patients with moderate-to-severe allergic asthma with a mean IgE level of 1046 IU/mL (range, 23–10,979 IU/mL) had a complete response to omalizumab. These patients demonstrated improvements in GETE scores, lung function, and asthma control, and >50% reduction in asthma exacerbations, corticosteroid steroid use, emergency room visits, and hospitalizations.^27^

In an analysis of the Severe Asthma Molecular Phenotype cohort from Paris in children (n = 45) with multiple allergic comorbidities and high total IgE levels (mean 1871 IU/mL; n = 18), omalizumab was found to be highly effective with a complete and rapid response in terms of improvement in asthma control, lung function, and reduction in frequency of severe exacerbations by more than 50%.^51^

The effectiveness of omalizumab treatment in these studies involving patients outside the dosing table range (>1500 total IgE) was almost in line with the efficacy/effectiveness observed in patients within the dosing table range. However, it should be taken into consideration that the number of patients with IgE >1500 IU/mL in which effectiveness of omalizumab was assessed is low compared with the overall population of patients. It is noteworthy that none of the studies involving patients with high IgE have reported a lack of response or any specific safety issues related to omalizumab. Overall, these findings were observed in patients with only high IgE levels and in those with both high IgE levels and high body weight, highlighting the efficacy of omalizumab in blocking early asthmatic responses over a broad range of IgE/body weight combinations and downregulating airway inflammation (as evaluated in terms of reduction in fractional exhaled nitric oxide levels, a marker for airway inflammation).

### Evidence for efficacy of omalizumab in patients with asthma and other allergic diseases

IgE levels can also reach extremely high levels (>1500 IU/mL) in other diseases that may easily overlap with asthma, which we call co-morbidities or multi-morbidities like atopic dermatitis, allergic bronchopulmonary aspergillosis (ABPA), allergic rhinitis, food allergy and nasal polyposis.^13^ IgE levels may reach >70 000 IU/mL in patients with atopic dermatitis^53^ and >24 000 IU/mL in those with aspergillus-sensitized asthma.^54^

Evidence from various clinical trials and case reports has demonstrated the efficacy and safety of omalizumab in patients with ABPA.^55^ In an Australian randomized controlled trial, omalizumab safely and effectively reduced IgE levels in patients with ABPA (mean baseline IgE levels: 2314 ± 2125 IU/mL).^56^ The efficacy and safety of omalizumab in adult patients with cystic fibrosis and difficult-to-treat ABPA was evaluated in a retrospective observational study in the Canadian population with median IgE of 889 IU/mL (715.5–2991.5 IU/mL). Lung function improved after initiating omalizumab in these patients and the treatment was well-tolerated.^57^

Omalizumab may also be effective in treating patients with atopic dermatitis (off-label use).^58^^,^^59^ In the atopic dermatitis *anti*-IgE pediatric double-blind, placebo-controlled randomized clinical trial (ADAPT), children with atopic dermatitis with median total IgE levels 8373 IU/mL (4556–18506 IU/mL) were treated with omalizumab or placebo for 24 weeks. Of note, 95% of children had baseline total IgE levels >1500 IU/mL. The study reported that children treated with omalizumab showed decrease atopic dermatitis severity and improve QoL despite elevated total IgE levels at baseline.^60^

Food allergy is frequently associated with allergic asthma and high IgE levels (up to ∼5000 IU/mL),^61^^,^^62^ representing a large unmet medical need, especially in the pediatric population. Omalizumab has been used off-label to treat food allergies and the National Institutes of Health is currently conducting a large clinical study (OUtMATCH) to confirm the efficacy of omalizumab in this population.^51^ Studies are also being conducted in Denmark to assess the efficacy of omalizumab in patients with food allergy.^63^

### Role of superantigen mediated increase in IgE levels

The increase in IgE levels in patients with allergic diseases is largely attributed to allergen sensitization. Recent reports suggest that microbial antigens such as enterotoxins from *Staphylococcus aureus* may lead to increased IgE levels through clonal amplification of IgE positive B cells and sensitization of basophils and mast cells.^64^^,^^65^ This superantigen mediated increase in IgE levels (serum IgE or IgE specific to *S. aureus* proteins) may have a potential role in the pathogenesis of asthma, and especially in late-onset asthma, and are associated with disease severity and exacerbations.^37^^,^^66^, ^67^, ^68^, ^69^ *Staphylococcus* superantigens have also been linked to elevated IgE levels (reaching up to 42 000 IU/mL) and increased severity in patients with atopic dermatitis^70^ and nasal polyposis,^66^ suggesting a strong role of a pathogen-driven increase in IgE levels in the etiology of chronic airway diseases.

### Role of microbial infections in asthma and other conditions with elevated serum IgE levels, with or without association with asthma

Sensitization against mold allergens such as Aspergillus, Alternaria, Cladosporium, Penicillium, and Candida is associated with more severe asthma phenotype requiring multiple hospitalizations.^71^ Moreover, infections caused by respiratory virus (particularly rhinovirus) and bacterial pathogens such as *Mycoplasma pneumoniae* and *Chlamydia pneumoniae* are known to be associated with increased asthma exacerbations.^72^ On the other hand, although the role of helminthic infections in either altering the allergic reactions depends on the intensity of the infection and the allergic condition of the subject, the presence of parasitic (helminthic) infections should be carefully reviewed as both helminthic infections and asthma are associated with elevated serum IgE levels.^73^

### Safety of omalizumab in patients with asthma with high IgE levels

The safety of omalizumab was also evaluated in patients with SAA with IgE >1500 IU/mL and none of the studies have reported safety issues specifically associated with high IgE levels^25^, ^26^, ^27^^,^^29^, ^30^, ^31^, ^32^, ^33^^,^^51^

In the eXpeRience registry (n = 925), patients with asthma with baseline IgE levels 8–7670 IU/mL were treated with omalizumab (as per labelling instructions) for 2 years. Although few serious adverse events (SAEs) were reported, the number of SAEs related to omalizumab was considerably low. Moreover, of nine deaths reported in the registry, none of them were suspected to be related to omalizumab. Overall, no unexpected safety events were reported.^30^

In a retrospective study (n = 52; 26 in each group), patients with asthma with mean IgE 221 IU/mL (Group 1) received omalizumab at a mean dose of 426 mg/month (range: 150–900 mg/month), whereas patients with mean IgE 2371 IU/mL (Group 2) received a mean dose of 886 mg/month (range: 400–1200 mg/month) for 6 months or longer duration. No patient reported anaphylaxis or severe injection site reactions. The study results showed that administration of omalizumab outside the therapeutic range showed comparable adverse events (AEs) between Group 1 and Group 2 patients.^26^

In a randomized study (omalizumab: n = 34; placebo: n = 16), asthmatic patients with IgE 30–300 IU/mL (Group 1) and IgE 700–2000 IU/mL (Group 2) were treated with omalizumab (as per dosing table) or placebo for 12–14 weeks. Overall, both omalizumab and placebo were well-tolerated with majority of AEs being mild to moderate in intensity. Nasopharyngitis was the most frequent AE reported. Severe malignant melanoma (Group 2) and breast papilloma (Group 1) were the serious AEs reported, but were not related to study drug. Two patients from the placebo group experienced severe AEs; none of these events were considered study drug related.^25^

In a 6-month analysis of Australian Xolair® Registry (n = 179), patients with asthma were grouped based on the dosing table (IgE levels and body weight) into within range and above range and were treated with omalizumab at a maximum dose of 750 mg/month. One patient from each of the groups discontinued omalizumab due to anaphylaxis.^28^

In the PROSPERO study, patients with asthma (IgE levels 1.1–68,628 IU/mL) were treated with omalizumab at a dose of 150 mg every 4 weeks (IgE<30 IU/mL) to 450 mg every 2 weeks (IgE >700 IU/mL) for a median duration of 11.2 months. Of 801 patients who received ≥1 dose of omalizumab, 90 patients experienced 144 S AEs, with the most common SAE being asthma (n = 26/801) and pneumonia (11/801). Although 7 deaths were reported, none of them were suspected to be study drug related. A total of 10 anaphylactic reactions were reported in 4 patients; 3 patients permanently discontinued the drug.^31^

In a case series conducted in children aged ≥6 years with IgE levels 892–4320 IU/mL (n = 11), omalizumab treatment (maximum dose: 375 mg every 2 weeks) resulted in no severe reactions. A single patient reported headache and dizziness; however, the patient continued treatment without any further AEs.^33^

In the REALITY study (n = 198), asthmatic patients with IgE levels 29–10979 IU/mL who were treated with omalizumab (maximum dose: 600 mg every 2 weeks) for a mean duration of 2.49 years showed no serious AEs.^27^

Overall, omalizumab has been shown to be well-tolerated in patients with SAA with IgE >1500 IU/mL.^25^, ^26^, ^27^, ^28^^,^^30^ In most of the studies, no deaths were reported.^25^^,^^27^ In some studies although few deaths were reported, they were not suspected to be drug related.^30^^,^^31^ Of note, the maximum dose of omalizumab administered in any of these studies is 600 mg every 2 weeks, which is in line with the omalizumab EU label recommendations;^74^ no unexpected safety signals were observed.^25^, ^26^, ^27^, ^28^, ^29^, ^30^, ^31^, ^32^, ^33^^,^^51^

## Discussion

The evidence included in this review confirms the therapeutic efficacy and effectiveness of omalizumab in patients with IgE levels >1500 IU/mL. The pharmacokinetics, pharmacodynamics and safety of omalizumab in patients with IgE levels up to 1500 IU/mL, treated based on the expanded dosing table in 2010, was also found to be comparable to the initially approved dosing table, potentially supporting the tolerability of omalizumab in patients with IgE levels >1500 IU/mL.^46^ In addition, the safety profile of omalizumab has been well-characterized based on the evidence from more than 20 years of real-life data, and an exposure rate surpassing 1.86 million patient-years,^42^ thereby supporting the potential for extending the dosing range to include patients with high IgE levels >1500 IU/mL.

It should be acknowledged that the studies discussed in this paper were identified following a comprehensive literature search based on search terms of English-language publications; the omission of relevant studies that may not have fit our specific search criteria cannot be discounted. Nevertheless, the evidence from the 9 studies surveyed in this manuscript, which included >3000 patients, support the potential therapeutic benefits of omalizumab in patients with severe asthma with IgE beyond the dosing range. Furthermore, it would be beneficial to conduct a study with different doses of omalizumab in patients with IgE levels >1500 IU/mL to provide more real-world evidence on effectiveness of omalizumab in this population.

### Potential basis for clinical outcomes in omalizumab-treated patients with high IgE levels and considerations during treatment

The efficacy of omalizumab in patients with IgE levels above the limit of the current dosing table, may be supported by its biological activity. Recent studies suggest that in addition to its affinity for free IgE, omalizumab in concentrations >1 μM may also detach IgE from the IgE:FcεR1 complex on basophils, in the absence of Cε2 domain IgE. This may in turn account for the lowered expression of FcεR1 on effector cells in patients treated with omalizumab.^75^ This sequestering effect has also been observed with IgE bound to mast cells in samples derived from patients with chronic urticaria,^76^ with similar mechanisms proposed for the treatment effect of omalizumab in asthma.^76^ Another potential mechanism is that at high concentrations, omalizumab destabilizes the interaction between IgE and FcεR1, promoting detachment of IgE from the receptor.^76^ These findings were based on *ex vivo* data and further investigation is needed to understand their relevance physiologically. Evidence from a recent *in vitro* study demonstrated that pre-treatment of plasmocytoid dendritic cells with omalizumab may restore activation of Treg cells, reducing the levels of pro-inflammatory cytokines and supporting extended clinical benefits with this *anti*-IgE biologic.^77^ Chang et al suggested a mechanism whereby the IgE:omalizumab complex is able to bind to additional allergens, blocking their interaction with receptor bound IgE and consequently preventing sensitization of mast cells and basophils. This would in turn serve as a feedback loop, preventing stimulation and release of allergen-specific IgE by B cells.^78^

Another important consideration for use of omalizumab in patients with asthma with high IgE is the variability in IgE levels over time. Patients with high IgE ineligible based on the dosing table may face increased risk of exacerbations, hospitalizations, and potential side effects from long-term use of OCS. A recent prospective study by Louis and colleagues^79^ showed that 24% of patients had IgE levels >700 IU/mL (range 729–7620 IU/mL) for ≥1 one visit, with a potential correlation with the change in asthma symptoms. The study suggests that 30% of patients ineligible for omalizumab based on initial IgE measurement may qualify after repeated measurements.^79^ Similar findings were also reported in an earlier study by Hatipoğlu and colleagues.^24^ Moreover, in a post-hoc analysis from the ICATA study, patients with persistent allergic asthma with IgE levels 30–1300 IU/mL were treated with omalizumab. This study suggests that patients with asthma who were considered suitable for treatment with omalizumab based on the disease severity, were considered ineligible as IgE levels were >1300 IU/mL.^80^

The fluctuation in IgE levels may present a further concern for accurate dosing of patients. The initial dosing levels of patients are determined based on baseline IgE levels; however, as treatment progresses, there may be changes in IgE levels, which may lead to over- or under-dosing. When treatment with omalizumab seems to be less effective in terms of asthma control, monitoring of IgE levels may help tailor treatment and determine whether the patients are receiving the appropriate dosing.^81^

**Fig. 3:**
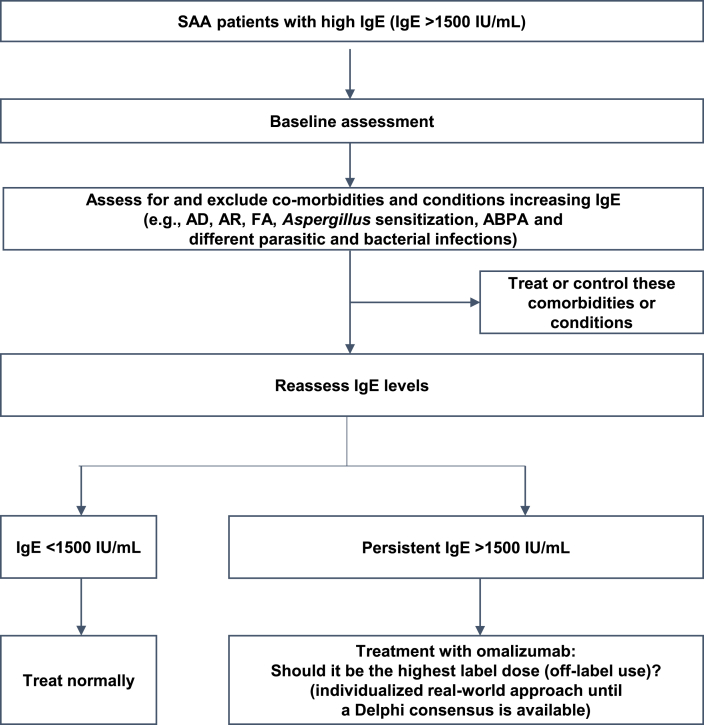
Management algorithm in SAA patients with IgE >1500 IU/mL. ABPA, allergic bronchopulmonary aspergillosis; AD, atopic dermatitis; AR, allergic rhinitis; FA, food allergy; IgE, immunoglobulin E; SAA, severe allergic asthma

Patients with SAA should be assessed at baseline for IgE levels before initiating treatment, and those patients with IgE levels >1500 IU/mL should be evaluated for any comorbidities or any microbial infections that may cause an increase in IgE levels. If there are any comorbidities or infections diagnosed, they should be treated first and then IgE levels should be reassessed. If the patients report persistent high IgE levels, treatment with omalizumab (may be, the highest dose) should be initiated. As the dosing schedule currently gives recommendations on dosing regimen for omalizumab only in patients with IgE ≤1500 IU/mL, Delphi consensus may be required to decide the dose in patients with IgE >1500 IU/mL (Fig. 3). In line with a recent roadmap proposed for eosinophilic asthma,^82^ the authors suggest that in patients with SAA and high IgE who show an initial partial response to omalizumab after 4 months, treatment can be continued with or without dose adjustments for another 6 months. Further assessments and a decision to continue or discontinue treatment should then be performed after a year of therapy. In addition, non-responders with very high IgE (for example >5000 IU/mL) should be assessed to confirm alternative phenotypes, or a potential diagnosis of other associated comorbidities should be considered.

Omalizumab has shown to be effective in patients with other comorbid allergic conditions such as ABPA, food allergy, nasal polyposis, etc., in whom high IgE levels (IgE >1500 IU/mL) were reported; hence, they should be taken into consideration during the therapeutic management of allergic asthma. Also, as high IgE levels may be mediated by concomitant bacterial and fungal infections,^83^^,^^84^ a potential approach in treating patients with high IgE could involve an initial search for these microbes and treatment with antimicrobial and antifungal agents. Anti-inflammatory treatment alone (topical calcineurin inhibitors, topical corticosteroids, ultraviolet therapies) reduces the colonization of *Staphylococcus aureus* in atopic dermatitis.^85^

### Summary and conclusions

High IgE levels (>1500 IU/mL) are observed in patients with SAA with or without an associated comorbid condition. Omalizumab has been shown in different studies to be an effective and well tolerated option in patients with a broad range of IgE levels reaching up to 67 000 IU/mL, with patients above the current dosing table range receiving comparable therapeutic benefit to patients within the dosing table range.

Extending the dosing table IgE range of omalizumab may potentially benefit SAA patients with IgE levels >1500 IU/mL, wherein there is a real and big unmet need. However, patients with IgE levels >1500 IU/mL should be evaluated for comorbidities and infection. If diagnosed, they should be treated first and then IgE levels should be reassessed. A detailed assessment of patients with high IgE levels is needed before deciding on the optimal treatment approach. A management algorithm for SAA patients with IgE >1500 IU/mL is proposed.

The maximum doses included in the current dosing tables in the European Union and the United States were the dose ranges validated in the clinical development program. After more than 20 years, many studies worldwide, with many patients having IgE levels >1500 IU/mL, showed effectiveness and a good safety profile for omalizumab with the doses in the current approved tables.

Will the highest dosing scheme be enough? Is there a necessity for assessment of new dosing? In the absence of dedicated phase III trials, will a Delphi methodology consensus be enough? Until then, a personalized approach is needed for each patient based on the existing data, the real-world practice and the remaining unmet medical needs. The medical need is out there, it is frequent and real!

## Abbreviations

ABP, allergic bronchoprovocative test; ABP, Aallergic bronchopulmonary aspergillosis; ACT, Asthma Control Test; ACQ, Asthma Control Questionnaire; AD, atopic dermatitis; AE, adverse event; AQLQ, Asthma-related Quality of Life Questionnaire; AR, allergic rhinitis; AUC, area under the curve; EMA, European Medicines Agency; ER, emergency roomEU, European UnionFA, food allergyFDA, Food and Drug AdministrationFeNO, fractional exhaled nitric oxide; FEV_1_, forced expiratory volume in 1 s; FVC, forced vital capacity; GETE, Global Evaluation of Treatment Effectiveness; ICS, inhaled corticosteroid; IgE, immunoglobulin E; LABA, long-acting beta-agonist; OCS, oral corticosteroid; SAA, severe allergic asthma; SAE, serious adverse event; SD, standard deviation; Th2, type 2 helper T cells; US, United States.

## Acknowledgments

The authors would like to acknowledge Dr. Roland Buhl for his valuable insights on this manuscript. Medical writing and editorial support for this manuscript was provided by Archana Jayaraman, Phani Tejasvi Dantu, Preethi B and Rahul Lad of Novartis Healthcare Private Limited, Hyderabad, India, which was funded by Novartis Pharma AG (Basel, Switzerland) in accordance with Good Publication Practice (GPP3) guidelines (http://www.ismpp.org/gpp3).

## Availability of data and materials

Not applicable as this is a review article and not an original research.

## Author contributions

All authors have contributed equally to the design, review, and preparation of the manuscript. All authors have contributed significantly to the work, have read the manuscript, attest to the validity and legitimacy of the data and its interpretation, and agree to its submission.

## Ethics statement

Not applicable, as this is a review article and not an original research.

## Authors’ consent for publication

I confirm that all authors have agreed to the submission and consequent publication of the manuscript, post-review by the editorial board.

## Declaration of competing interest

**Francesco Menzella** has received research grants from AstraZeneca, Novartis Farma, and Sanofi; and lecture fees and advisory board fees from AstraZeneca, Boehringer Ingelheim, Chiesi Farmaceutici, GSK, Mundipharma, Novartis Farma, Angelini, and Sanofi.

**Jocelyne Just** has received consulting fees from Novartis, Sanofi, ALK-Abello, and Astra Zeneca; advisory board and meetings/travel support from Novartis, Sanofi, ALK-Abello, and Astra Zeneca; lecture fees from GSK, Sanofi and Novartis; and grants from Novartis, Astra Zeneca and ALK-Abello.

**Inessa Schwab Sauerbeck, Claudia Mailaender, Fabiana Saccheri, Celine Thonnelier, Xavier Jaumont and Laurence Mala** are employees of Novartis.
